# Cardiomyocytes in Hypoxia: Cellular Responses and Implications for Cell-Based Cardiac Regenerative Therapies

**DOI:** 10.3390/bioengineering12020154

**Published:** 2025-02-06

**Authors:** Kiera D. Dwyer, Caroline A. Snyder, Kareen L. K. Coulombe

**Affiliations:** Institute for Biology, Engineering, and Medicine, School of Engineering, Brown University, Providence, RI 02912, USA; kiera_dwyer@brown.edu (K.D.D.); caroline_a_snyder@brown.edu (C.A.S.)

**Keywords:** hypoxia, myocardial infarction (MI), ischemia, heart regenerative, stem cell therapies, stem cell derived cardiomyocytes (SC-CMs), pro-survival

## Abstract

Myocardial infarction (MI) is a severe hypoxic event, resulting in the loss of up to one billion cardiomyocytes (CMs). Due to the limited intrinsic regenerative capacity of the heart, cell-based regenerative therapies, which feature the implantation of stem cell-derived cardiomyocytes (SC-CMs) into the infarcted myocardium, are being developed with the goal of restoring lost muscle mass, re-engineering cardiac contractility, and preventing the progression of MI into heart failure (HF). However, such cell-based therapies are challenged by their susceptibility to oxidative stress in the ischemic environment of the infarcted heart. To maximize the therapeutic benefits of cell-based approaches, a better understanding of the heart environment at the cellular, tissue, and organ level throughout MI is imperative. This review provides a comprehensive summary of the cardiac pathophysiology occurring during and after MI, as well as how these changes define the cardiac environment to which cell-based cardiac regenerative therapies are delivered. This understanding is then leveraged to frame how cell culture treatments may be employed to enhance SC-CMs’ hypoxia resistance. In this way, we synthesize both the complex experience of SC-CMs upon implantation and the engineering techniques that can be utilized to develop robust SC-CMs for the clinical translation of cell-based cardiac therapies.

## 1. Introduction

Oxygen is fundamental to human survival, originating from its molecular functions and permeating up to the whole organ and organismal levels. Oxygen plays a vital role in a variety of critical biochemical reactions that maintain intracellular adenosine triphosphate (ATP) levels, such as aerobic respiration and fatty acid desaturation, as well as other vital processes such as biosynthesis (e.g., collagen synthesis [1]), post-translational modification (e.g., protein hydroxylation [2]), and epigenetic regulations (e.g., RNA, DNA, and histone demethylation [3,4]). In these reactions, oxygen can serve as an electron acceptor, as in the case of aerobic respiration, to facilitate electron movement for ATP synthesis; or as an oxidant to catalyze reactions, as in the case of α-ketoglutarate dioxygenase reactions in biosynthesis [5]. Hypoxia is characterized by a reduced oxygen concentration and is a significant stressor to physiological homeostasis. Cellular hypoxia (<2% pO_2_) describes a mismatch between oxygen supply to the cell and its metabolic demand. The ability to understand how cells sense and adapt to hypoxic environments is incredibly important, as hypoxia plays a critical role in a variety of diseases that top the leading causes of death worldwide, such as cardiovascular disease, cancer, chronic kidney disease, and metabolism disorders [6]. Such importance is evidenced with the 2019 Nobel Prize in Physiology or Medicine being awarded to William G Kaelin Jr., Sir Peter Ratcliffe, and Gregg L. Semenza “for their discoveries of how cells sense and adapt to oxygen availability”.

In the context of the heart, hypoxia plays a key role in cardiovascular disease and its progression due to the high oxygen demand of the heart. Often, the heart is referred to “an obligatory aerobic organ”, as it cannot produce enough energy under anaerobic conditions to maintain the essential processes of cardiomyocytes (CMs). In this way, the heart consumes more oxygen per minute by mass than any other organ [7]. When the heart does experience hypoxia from disease and/or injury, it has a detrimental effect on the ability of the heart to function due to the failure of many cellular processes within the CMs. In this way, understanding the response of CMs to hypoxia is vital to developing therapies that mitigate this damage and preserve the function of the heart.

## 2. Hypoxia in the Heart

Although hypoxia is typically considered to have detrimental impacts on cell function, it is important to acknowledge its role in healthy heart function. Interestingly, hypoxia plays a vital role in utero, with the majority of fetal heart development occurring in relative hypoxia, which is hypothesized to modulate trophoblast differentiation and invasion [8,9], as well as allow for proper outflow development [10] and coronary artery formation [11]. Further, the heart has a considerable capacity to tolerate and mitigate mild hypoxia through physiological adaptations, such as at high altitudes. In acute altitude hypoxia exposure (i.e., 0–12 h) for people not accustomed to altitude, the physiological changes of the heart are focused on increasing cardiac output (CO) facilitated by increased heart rate (HR) (i.e., CO = HR × SV) to sustain systematic oxygen delivery (i.e., O_2_ delivery = arterial O_2_ content × CO) [12]. As exposure is prolonged, hypoxia induces pulmonary vasoconstriction, increased hemoglobin concentration, and increased left ventricle (LV) torsion [12,13,14]. Populations native to high altitude, however, exhibit generational cardiovascular adaptions including increased HR; mild right ventricle (RV) hypertrophy (likely due to chronically elevated pulmonary pressure); smaller LV volumes; increased hemoglobin mass; and higher total blood volumes [15,16,17].

In many instances, however, hypoxia reaches a level above which the human body can no longer compensate for, such as a myocardial infarction in the heart. Myocardial infarction (MI), also known as a heart attack, is a well-known cardiac event characterized by reduced or loss of blood flow to part of the myocardium. It is important to note, here, the connection between ischemia and hypoxia, with ischemia being defined as a reduction in blood flow to a given area of tissue while hypoxia refers to a reduction in the oxygen level, causing an imbalance between oxygen supply and demand [18]. In this way, MI causes ischemia (i.e., loss of blood flow) which results in severe hypoxia due to the prolonged deprivation of oxygen from the myocardium [19]. A hallmark of MI in heart attack patients is large-scale cell death—up to one billion CMs over the course of minutes to hours [20].

### 2.1. Pathophysiological Impact of MI on CMs

**Figure 1:**
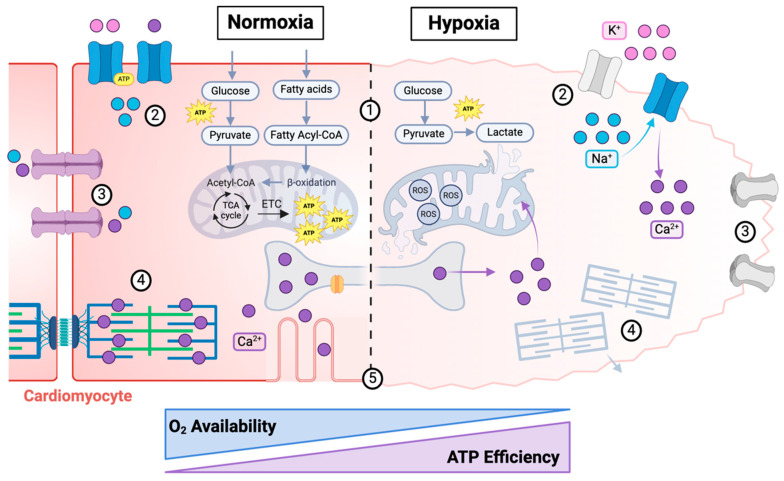
**Summary of cellular changes that occur within cardiomyocytes (CMs) in hypoxia during myocardial infarction (MI)**. (1) CM metabolism switches from aerobic pathways (fatty acid oxidation and mitochondrial respiration) to anaerobic pathways (glycolysis), impacting multiple parallel cell systems. (2) Ion channels begin to fail, causing increased intracellular sodium concentration and increased extracellular potassium concentration, impacting (3) the electrical activity of the CMs, including gap junctions. (4) CM contractility decreases due to altered calcium buffering, disruption of sarcomeres, and reduced ATP availability. (5) CM morphology also changes due to myofibril stretching, cell swelling, and depletion of glycogen stores. Created in BioRender. Dwyer, K. (2025) https://BioRender.com/w84n608 (accessed on 3 January 2025).

**Figure 2:**
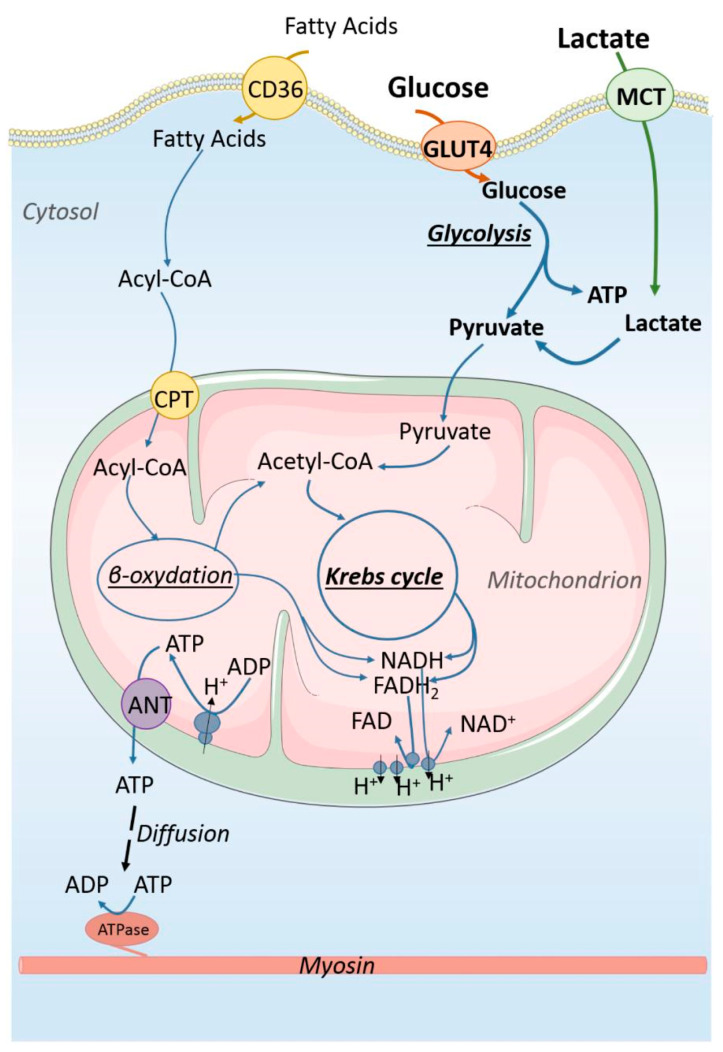
**Metabolism of cardiomyocytes (CMs) under normoxic conditions**. In healthy CMs, the majority of energy is produced through the oxidative phosphorylation of fatty acids and glucose. In high concentrations, lactate can be utilized in energy production through its conversion to pyruvate. CD36 is the fatty acid translocase; GLUT4 is the glucose transporter 4; MCT is the monocarboxylate transporter; Acyl-CoA is acyl-coenzyme A; NAD and NADH refer to oxidized and reduced nicotinamide adenine dinucleotide; and FAD and FADH_2_ refer to oxidized and reduced, flavin adenine dinucleotide. Image reproduced from [37], copyright © 2018 Piquereau and Ventura-Clapier under the Creative Commons Attribution License (CC BY).

MI triggers a complex cascade of events impacting cellular signaling, tissue function, and overall organ remodeling. As in other diseases, cellular injury is the basis of MI. In response to hypoxia, CMs instigate compensatory mechanisms and experience dysfunction in metabolism; ion imbalances and calcium handling; electrical activity; contractility; and morphology. A summary of the complex changes that occur within CMs in hypoxia during MI is depicted in Figure 1 and will be the focus of this section. In addition to exploring the molecular processes underlying MI injury within CMs in key functions, this section will explore their interconnected nature as well as how changes at the CM level influence the overall function and environment of the heart (Figure 2).

#### 2.1.1. Metabolic Changes

The heart is very energy-demanding to support the continuous contractile cycling of CMs that drive its pumping. Because of this, the heart has the highest uptake of oxygen (~0.1 mL O_2_/g/min) and energy requirements (~6 kg of ATP per day) of all the organs in the human body [18,21]. To accommodate this high energy demand, CMs have robust metabolic processes as illustrated in Figure 2. In the healthy human heart, it is estimated that 95% of ATP is produced from glucose and fatty acid metabolism [18]. Acetyl-CoA, which is formed from glucose through the conversion of pyruvate in glycolysis by the pyruvate dehydrogenase (PDH) complex, and from fatty acids through β-oxidation, undergoes oxidative phosphorylation (i.e., the citric acid cycle or tricarboxylic acid cycle, TCA, coupled with an electron transport chain, ETC) (Figure 2). The ATP generated from the oxidation of fatty acids is significantly higher compared to glucose (106 vs. 32 molecules of ATP per molecule of palmitate vs. glucose, respectively), yet, it is actually less efficient in terms of oxygen consumption [18,22]. It has been calculated that the oxidation of 1 mol of glucose generates 53.7% more high-energy phosphate bonds per O_2_ compared to fatty acids [18,23]. The shift from fatty acid toward glucose metabolism has been well documented in chronic hypoxia conditions, with enhanced glucose uptake in the myocardium and lower plasma glucose in chronically hypoxic populations compared to lowlanders [24].

Within the context of MI, in early hypoxia (~seconds to minutes), the flux of substrate into the TCA cycle does not change [25]. Yet, prolonged hypoxia induces changes in CM metabolism which are modulated primarily through the activation of a family of transcription factors known as the hypoxia-inducible factors (HIFs). There are three HIF isoforms, HIF-1, HIF-2, and HIF-3, each of which have α and β subunits, with the most studied isoform being HIF-1 [26]. In the heart, HIF-1α and HIF-1β are ubiquitously expressed, but their stability is differentially regulated by the oxygen level (i.e., HIF-1α is degraded in normoxia and HIF-1β is constitutively stable), making them potent oxygen sensors. In normoxic conditions, HIF-1α subunits are hydroxylated by prolyl hydroxylase domain-containing enzymes (PHDs) and marked for degradation by proteosomes [26]. Hypoxic conditions result in reduced PHD activity and the stabilization of HIF-1α. HIFs are transcriptional regulators, so when stabilized in hypoxia, they induce several important downstream effects, which we discuss below as they relate to stem cell-derived cardiomyocytes (SC-CMs) for regenerative medicine, such as the metabolic shift of CMs from oxidative phosphorylation to glycolysis. It is estimated that within 15–20 s of hypoxia, glycolysis becomes the only significant source of ATP for the CMs [27].

The specific mechanisms surrounding this metabolic shift in hypoxia have been well studied in adult CMs. First, it has been reported that HIF-1 signaling limits the input material to oxidative phosphorylation by driving the pyruvate conversion to lactate, rather than acetyl-CoA, through the upregulation of two targets: (1) lactate dehydrogenase A (LDH-A), which converts pyruvate to lactate and increases NAD+ to drive glycolysis, and (2) pyruvate dehydrogenase kinase 1 (PDK1), which phosphorylates and inactivates PDH to drive the pyruvate conversion to lactate [25]. Further, the activity of the ETC is also reduced by both hypoxia directly and HIF-1 signaling. For example, oxygen (<0.3%) becomes a limiting factor for cytochrome C oxidase (COX, complex IV of the ETC), the main enzyme which utilizes oxygen to synthesize ATP in the ETC, thus slowing its enzymatic rate [25]. Further, in low oxygen conditions, nitric oxide (NO), which is a competitive inhibitor of O_2_ to COX, increases to inhibit COX activity [28,29]. HIF-1 signaling, additionally, has been implicated in targeting inducible NO synthase (iNOS) to increase NO levels [30,31]; increasing micro-RNA 210 levels which inhibits the function of the ETC through complex I, COX subunit 10, aconitase, and subunit D of succinate dehydrogenase; and inducing the COX subunit switch to optimize metabolic efficiency [32].

The reliance of ATP production on glycolysis within the CM during prolonged hypoxia leads to a cascade of events that result in: (1) the depletion of intracellular glycogen stores utilized in glycolysis due to the lack of perfused glucose; (2) intracellular acidosis due to the accumulation of lactate in the cytoplasm caused by the reduction of pyruvate (a by-product of glycolysis) to lactate, as opposed to its transport to the mitochondria for oxidative phosphorylation; and (3) the subsequent inhibition of several enzymes related to glycolysis caused by acidosis resulting in decreased rates of glycolysis after 15–20 min of hypoxia [33].

Even at its fastest rate, glycolysis cannot keep up with the energy demands of CMs. It is estimated that ATP content is depleted by 65% after 15 min of ischemia, and by 90% after only 40 min [34]. Because many of the processes within the cell require energy, ATP deficiency has a profound negative impact on several other aspects of CM function which are described in the following sections. First, CMs experience the disruption of ion homeostasis due to the dysfunction of several energy-dependent pumps. Such an ion imbalance impacts the ability of the CM to maintain the action potential, regulate calcium, and propagate an electrical signal. From a mechanical point of view, sarcomeres become dysfunctional, losing the ability to contract or relax, as both are energy-dependent processes. Finally, the ATP-dependent processes of mRNA translation [35,36], organelle structure, and junction maintenance are inhibited.

#### 2.1.2. Ion Balances and Calcium Accumulation

The ATP-depleted environment of the heart in hypoxia causes a severe ion imbalance within CMs through the dysregulation of ATP-dependent ion pumps. One of the most significantly impacted pumps is the Na^+^/K^+^ ATPase, which requires ATP to exchange intracellular Na^+^ for extracellular K^+^ against their concentration gradients, as it is estimated to use 20–50% of the energy expenditure of muscle cells [38]. Thus, during hypoxia, there is an efflux of intracellular K^+^, causing accumulation in the extracellular space, which has a detrimental functional impact on CM excitability. At the same time, intracellular Na^+^ levels rise due to the reduced function of the H^+^/Na^+^ exchanger (HNX) [39]. Increased Na^+^ intracellular levels in turn increase the activity of the Na^+^/Ca^2+^ exchanger (NCX) to excrete Na^+^, resulting in intracellular calcium accumulation [39]. Therefore, during hypoxia, the intracellular calcium level increases slightly due to this accumulation coupled with the reduced ATP levels of the CMs, which inhibit the function of ATP-dependent calcium pumps such as sarco/endoplasmic reticulum Ca^2+^-ATPase 2a (SERCA2a), which sequesters Ca^2+^ back into the sarcoplasmic reticulum (SR) through the hydrolysis of ATP [40]. During reperfusion, however, calcium levels within the CM increase to a point of inducing irreversible cellular damage, a phenomenon known as “calcium overload”, which will be discussed below.

#### 2.1.3. Electrical Activity and Arrhythmia Generation

One of the major consequences of hypoxia-induced ion imbalances within CMs is electrical dysfunction. Cardiac electrophysiology is based on the generation of an action potential (AP) by individual CMs, which is a measure of the voltage changes across the membrane of a cell. Under normoxic conditions, ion channels throughout the cell membrane change their permeability to drive ion movement and thus change the membrane potential of the cell [41]. Because of this, ion homeostasis maintained by ATP-dependent pumps is vital. In hypoxia, such homeostasis is perturbed, which in turn impacts the ability of the CM to generate an AP. For example, the efflux of K^+^ into the extracellular space during hypoxia leads to a reduction in AP amplitude and the rate of AP rise [42]. Prolonged K^+^ accumulation further results in CM in-excitability and conduction block [33]. Additionally, intracellular acidosis has been implicated in the inhibition of most ion channels, resulting in a depolarized resting potential, the prolongation of AP duration, and an increased early afterdepolarization occurrence [33,43].

**Figure 3:**
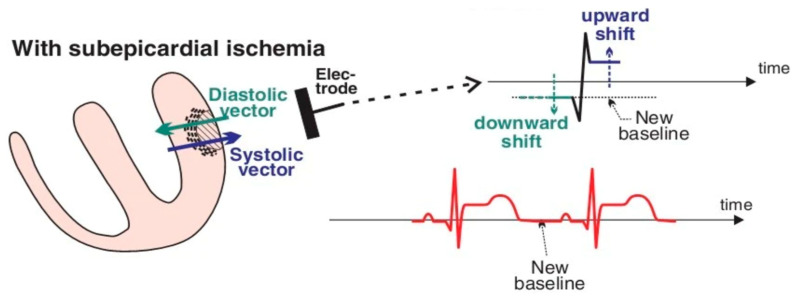
**Schematic of ST displacement in ischemic myocardium post-MI captured by electrocardiogram (ECG)**. An apparent elevation in ST occurs in transmural or epicardial ischemia due to the early depolarization of the infarcted myocardium. Image reproduced from [53], copyright © 2016, John Wiley and Sons with permission.

The disruption of electrical activity at the cellular level has important repercussions for overall cardiac function. Approximately 90% of patients develop rhythm disturbance post-MI, with the greatest risk peaking in the first hour post-MI and declining thereafter [44,45]. Such MI-associated arrythmias can include bradyarrhythmia, atrioventricular (AV) block, supraventricular tachyarrhythmias, as well as life-threatening ventricular arrhythmias (VA) [46,47]. Importantly, the electrical dysfunction of CMs throughout the heart can be measured through skin electrodes to capture the electrocardiogram (ECG), which serves as an important tool for emergency diagnosis and treatment. There are three changes to the ECG, rooted in cellular changes in hypoxia, which clinicians use to diagnose MIs and make treatment decisions for patients:

(1) **ST-segment displacement**: Electrocardiography is used to classify MIs into ST-segment elevation MI (STEMI), in which there is prolonged ST-segment displacement towards more positive membrane potentials on the ECG; or non-ST-segment elevation MI (NSTEMI). Despite having a similar clinical presentation, STEMIs often result from the total occlusion of the coronary artery and current management strategies advise immediate reperfusion, while NSTEMIs indicate the incomplete, yet critical occlusion of the coronary artery [48]. Connecting this global electrical signal from the heart to the cellular level, the healthy ST segment of an ECG represents the isoelectric period between ventricular depolarization and repolarization, in which there is no change in membrane voltage. Due to the failure of the Na^+^/K^+^ ATPase pump from ATP deficiency within hypoxic CMs and the efflux of K^+^, early depolarization spreads to the adjacent uninjured myocardium, thus elevating the isoelectric point of the tissue between depolarization and repolarization (i.e., the ST segment). For transmural infarcts (which are the most severe), the early depolarization that results in an elevated isoelectric point is captured by an apparent ST-segment elevation on an ECG, as shown in Figure 3. Further, computational work in the field also implicates the loss of AP plateau amplitude from the CMs in the epicardial region [49], as well as the functional decline of Na^+^ and ATP-sensitive K^+^ channels, in directly displacing the ST-segment of the ECG during ischemia [50].
(2) **Pathological Q-wave**: The Q-wave on a healthy ECG represents the left-to-right depolarization of the interventricular septum. A pathological Q-wave, which is defined as being wider (>40 ms) or deeper (>2 mm or >2 mV) compared to healthy patients, indicates the absence of electrical activity and thus is a sign of myocardial necrosis. In the context of MI, pathological Q-waves typically occur 6–16 h after the initial onset of ischemia, when the infarcted region of myocardium ceases its electrical activity. Although ST elevation is the first determination of MI and drives most of the treatment decisions by physicians, the prognosis of Q-waves provides an additional tool of infarct timing and severity without additional costs or delays [51]. In a clinical study, both subendocardial (28%) and transmural (29%) MIs showed pathological Q-waves, with MI size and the number of transmural segments increasing the pathological Q-wave probability [52].
(3) **T-wave inversion**: The T-wave represents the transmural dispersion of repolarization. In the healthy heart, repolarization occurs from the epicardium to the endocardium. However, in ischemia, which first impacts the endocardial region, this repolarization direction is switched due to the K^+^ extracellular accumulation and shortening of the AP duration in the infarcted endocardium, causing T-wave inversion on the ECG. Although T-wave inversion is not used by itself to diagnose MI, it can be used by clinicians to determine the location of infarct based on which areas of the heart feature T-inversion as well as which coronary arteries may be blocked.

#### 2.1.4. Contractility and Mechanical Compensation

The sarcomeres within CMs are responsible for the contraction and relaxation of the heart during the cardiac cycle. It has been estimated that within 60 s of artery occlusion, the contractile force generation of CMs is suppressed [54,55], which can be explained by the following mechanisms:

1. **ATP deficiency.** The cyclic binding of cardiac myosin to actin requires splitting ATP for the energy to generate force and to shorten, followed by the binding of a new ATP molecule to release myosin from actin. With prolonged ATP deficiency, sarcomeres begin to lose the ability to generate force and shortening, and relaxation is inhibited, effectively causing a slowing of actomyosin cycling. Although the metabolic switch to glycolysis is able to meet the basic energy needs of the CMs, including some contraction, the energy depletion and demand become too great within 60–90 min and CMs undergo contracture rigor [56].
2. **Inhibition, modification, and degradation of contractile proteins**. It has been shown that contractile protein composition (myosin, actin, troponin isoforms, etc.) does not change in hypoxia [57]; rather, contractile protein function is inhibited. During hypoxia, there is an accumulation of inorganic phosphate due to the rapid breakdown of ATP reserves. The high level of inorganic phosphate within the CMs, which rises from 2 to 5 mM to as much as 15–20 mM in hypoxia, has been shown to reduce the contractility [58,59]. In one study, 20 mM inorganic phosphate reduced the maximum calcium-regulated force of cardiac muscle by as much as 69% [59]. Additionally, increased levels of reactive species during hypoxia induce chemical modifications of sarcomeric proteins, which in turn alter their structure and reduce their functional contractile activity [60]. Such chemical modifications include the oxidation (typically occurring on cysteine, Cys) and nitrosylation (typically occurring on tyrosine, Tyr) [60] of several sarcomeric proteins, including cardiac troponin I (cTnI) and cardiac troponin T (cTnT) [61,62]; tropomyosin [63,64,65,66,67]; myosin heavy chain (MHC) [68,69,70]; myosin light chain (MLC) 1 [61,71] and 2 [72]; α-actinin [61,62,73]; and titin [74].
3. **Calcium handling dysfunction.** Calcium is a vital signaling molecule in cardiac contractility, as it links electrical activation with mechanical contraction in the heart [75]. In hypoxia, APs and calcium transients are preserved in the early phases, so initial contractile dysfunction is largely attributed to the inhibition of contractile proteins [33]. In vitro studies have shown that intracellular acidosis decreases calcium binding to sarcomeric proteins, importantly, to troponin C, thus inhibiting contractility [76,77]. Further, ATP deficiency contributes to increased cytosolic calcium concentrations because ATP, which is depleted in hypoxia, is necessary to pump calcium against its concentration gradient back into SR [33].

At the organ level, the overall cardiac mechanics during MI are compromised. During the acute phase of MI, the infarcted region ceases its contraction and the regional mechanics of the infarct are dominated by the passive properties of the tissue [78]. As quickly as 30 s after coronary occlusion, systolic shortening is replaced by stretching with passive recoil of the infarcted region [79]. Reduced ejection fraction (EF, stroke volume/end-diastolic volume) is typical of post-MI patients, where a 10% reduction in EF is associated with a 39% increase in the hazard ratio for all-cause mortality [80]. Yet, if the highly acute ischemic injury is managed and the patient survives, the heart can undergo sophisticated remodeling, such as increasing end-diastolic and end-systolic volumes in order to maintain cardiac output (CO, stroke volume × heart rate). Such volume increases are associated with higher stresses, dilation, and increased compliance in the early MI stages, as evidenced by a decrease in the end-diastolic pressure–volume relationship (EDPVR) [81].

#### 2.1.5. Morphology and Blood Biomarkers

**Figure 4:**
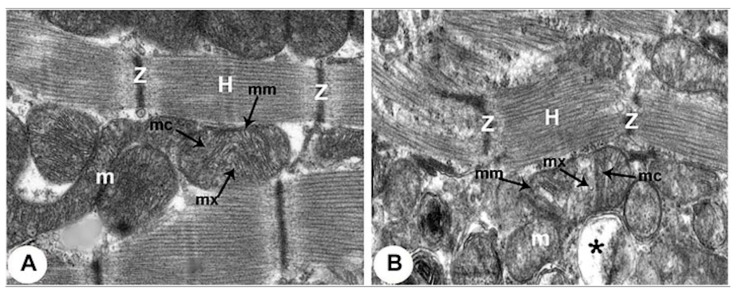
**Morphological disorganization that occurs within cardiomyocytes (CMs) during hypoxia.** Transmission electron micrographs (300,000X) of rat hearts in normoxic (**A**) and hypoxic (**B**) conditions. Hearts exposed to hypoxia display disrupted contractile elements (Z and H bands), mitochondrial (m) degeneration (*) (inner membrane, mm; matrix, mx; cristae, mc). Image reproduced from [83], copyright © 2022 Shati, Zaki, Alqahtani, Haidara, Alshehri, Dawood, and Eid under the Creative Commons Attribution License (CC BY).

In conjunction with the cardiac functional changes occurring during hypoxia, CM morphology is significantly impacted. The ion imbalance associated with CM hypoxia creates a hyperosmolar environment, causing the swelling of the CM due to an increased water uptake. Such swelling contributes to reduced compliance, dilation of the endoplasmic reticulum, and changes in membrane permeability [82]. Histological staining and electron microscopy have further revealed the impact of hypoxia of specific organelles within the CMs [83]. For example, the myofibrils, which contain the contractile proteins of CMs, become disorganized with fragmentation occurring within the structural muscle bands (Z and H bands), as illustrated in Figure 4. The membranes of the organelles have also been shown to deteriorate during hypoxia. First, the mitochondria become swollen, leading to breaks in the mitochondrial outer membrane. Then, in the nucleus, the chromatin becomes clumped and the nuclear envelope dissembles. Further, intercellular CM junctions become damaged, with intercalated disks (composed of fascia adherens, desmosomes, and gap junctions) becoming disorganized and disrupted in hypoxia.

Clinicians use biomarkers of CM breakdown due to hypoxia to confirm MI in patients. Within hours of the onset of MI, the plasma membrane of swollen CMs can rupture, releasing intracellular molecules or their fragments to the extracellular space, which can be measured from blood serum to assess MI [82]. For example, immunoassays have been developed to assess the levels of cardiac troponin T and I (cTnT, cTnI) fragments, proteins that regulate muscle contraction, that have leached from the necrotic myocardium into the bloodstream. Elevated levels of cTnT (within 2–3 h of MI onset; males: 15.5 nanogram/L, females: 9 nanogram/L) and/or cTnI (within 2–6 h of MI onset; males: 26 nanogram/L, females: 16 nanogram/L) indicate MI [84]. Further, because the apparent half-life of cTnT and cTnI in the plasma resulting from MI is ~24 h, this assay provides an acute diagnostic tool [85]. Although less cardiac specific than troponins, creatine kinase MB isoenzyme (CKMB) has also been used in immunoassays to detect MI and re-infarction [86].

### 2.2. Reperfusion Injury

**Figure 5:**
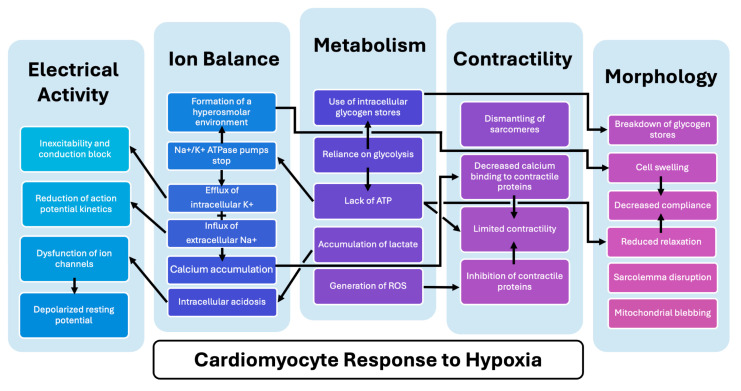
**Interconnected response of cardiomyocytes (CMs) to hypoxia during myocardial infarction (MI)**. CM properties, including metabolism (**middle**), ion imbalance (**middle-left**), electrical activity (**far left**), contractility (**middle-right**), and morphology (**far right**) impact each other, determining the overall function and survival of the cell.

While the restoration of blood flow (and thus oxygen supply) to the infarcted myocardium is the standard of care (i.e., thrombolytic therapy or primary percutaneous coronary intervention for balloon angioplasty and stenting) to reduce patient mortality, several responses at the cellular level discussed previously become exacerbated, resulting in further cell death. This phenomenon is known as myocardial ischemia/reperfusion injury (MIRI). Animal studies of acute MI suggest injury associated with reperfusion can account for up to 50% of the final infarct size [27]. For example, Jennings et al. found reperfusion accelerated CM death, equating cell damage from 30 to 60 min of MI with reperfusion to 24 h of permanent occlusion, as assessed through histological staining in a canine model [87]. The following mechanisms have been proposed during MIRI, which contribute to the interconnected nature of CM damage in hypoxia (Figure 5):

1. **Reactive oxygen species (ROS) generation**. ROS are molecules that contain oxygen and which are highly reactive, including but not limited to superoxide anion (O_2_^−^), hydroxyl radical (HO^−^), nitric oxide (NO), and hydrogen peroxide (H_2_O_2_). Because of their high reactivity, these ROS can cause oxidative damage throughout the cell through the oxidation of macromolecules such as DNA, RNA, lipids, and proteins, which may lead to cell death [88]. During active hypoxia, ROS can be produced in the mitochondria within the ETC, specifically complexes I and III, where electron leakage occurs resulting in O_2_ formation. However, it is well documented that ROS generation is significantly increased during reperfusion with the influx of oxygen through a variety of mechanisms [89]. ROS are produced within the first minutes of reperfusion after MI [90], and much research in the heart has connected ROS to directly damaging DNA, proteins, and lipids throughout the CM [27].
2. **Rapid restoration of metabolism and pH**. With reperfusion and the influx of fatty acids, the metabolism of CMs shifts back to oxidative phosphorylation but overshoots. The increased fatty acid oxidation inhibits glucose oxidation, which results in the conversion of pyruvate derived from glycolysis into lactate, with a net production of two H^+^ [27]. To restore the pH from both glycolysis during hypoxia and the accumulation of intracellular H^+^ from reperfusion, the H^+^/Na^+^ pump is activated, with H^+^ being pumped into the extracellular space while Na^+^ accumulates within the cell.
3. **Calcium overload**. In response to the increased intracellular Na^+^ levels, the Na^+^/Ca^2+^ pump is activated, increasing intracellular calcium levels and overloading the CM. With the availability of ATP and calcium, CMs may contract excessively and uncontrollably, a phenomenon known as hypercontracture [91]. Such contractile dysfunction risks damage to the sarcomeric and cytoskeletal proteins as well as the injuring of intercellular junctions. Further, a phenomenon known as “myocardial stunning” has been reported post-MI reperfusion in which the myofilaments of infarcted CMs experience decreased sensitivity to calcium, leading to prolonged, yet reversible, contractile dysfunction. Both ROS oxidation and the calcium-induced proteolysis of contractile proteins have been hypothesized as potential mechanisms of this injury, with the repair and synthesis of the damaged proteins occurring during the stunning period, thus allowing the CMs to eventually restore their contractile function [92].
4. **Mitochondrial damage**. The CM fate during reperfusion is largely attributed to the extent of mitochondrial damage. The mitochondrial permeability transition pore (PTP) is a nonselective channel within the inner membrane of the mitochondria which is sensitive to reperfusion injury. Both oxidative stress and calcium overload can trigger the opening of the mitochondrial PTP [93]. Once permeabilized, the mitochondrial membrane potential collapses and oxidative phosphorylation within the cell becomes uncoupled, resulting in ATP depletion and cell death [27,94].

## 3. Consequences of MI on the Cardiac Environment

MI is an acute cardiac event, yet the severe stresses it imposes on CMs initiates a flood of alterations to the myocardial tissue. Although the changes that occur at the cellular level during the hypoxia of MI are distinct (i.e., metabolism, ion imbalance, electrical activity, contractility, morphology), they are intrinsically interconnected, as illustrated in Figure 5. Such changes have long-lasting impacts on the environment of the heart. Even after the heart is reperfused, irreversible CM injury triggers changes in the cardiac environment in the days to weeks after the initial MI. Understanding the changes to cell viability, composition, extracellular matrix deposition, and persistent ischemia, among other environmental changes, are highly relevant in the development of cell-based therapies which aim to regenerate cardiac function by introducing healthy CMs to the post-MI cardiac environment. 

### 3.1. Cell Death

In hypoxia, CMs become noncontractile within 1–2 min of hypoxia but may not die for another 20–30 min [95]. During this time, sarcolemma breaks and mitochondrial structural damage are markers indicating irreversible damage to the CMs and their eventual death [33]. Importantly, several mechanisms of CM death in hypoxia have been proposed, each of which has unique implications for the cardiac environment.

The predominant mechanisms of cell death in MI are apoptosis and necrosis [96]. Apoptosis is defined as regulated cell death, characterized by cell shrinkage and fragmentation with eventual removal by phagocytes. Both the intrinsic (mitochondrial membrane permeabilization and the release of cytochrome C to trigger caspase activation) as well as extrinsic (death receptor) pathways of apoptosis have been reported in MI [96,97], both of which result in DNA fragmentation and do not illicit an immune response. On the other hand, necrosis is marked by cell swelling and membrane (sarcolemma and/or mitochondrial) rupturing, which releases cytosolic contents into the extracellular space to trigger an immune response. Although both apoptosis and necrosis are known to be involved in MI, the relative contribution of each is less known [96]. Further, several forms of regulated necrosis have been reported in MI, such as necroptosis (a caspase-independent pathway in response to sarcolemma tumor necrosis factor, TNF, or Toll-like receptors) [98] and pyroptosis (caspase-dependent pathway activated by the formation of inflammasome multiprotein complexes) [99], both of which are characterized by the loss of plasma membrane integrity.

Most of the CMs in the infarcted region that undergo cell death do so over the first 6–24 h post-infarct [100]; however, a second wave of CM death weeks to months post-MI can be observed due to activation of proinflammatory pathways, biomechanical stress, and/or continued ventricular dysfunction [33,101]. Such cell death causes several morphological changes to the environment of the heart, such as wall thinning, increased wall stress, and dysregulation of gap junctions, desmosomes, and adherens junctions in the border zone, making the heart highly arrhythmogenic [102].

### 3.2. Scar Formation and Immune Response

Cardiac remodeling continues during the wound healing and scar formation processes, which include the activation of a complex and dynamic immune response. Three phases are used to describe scar formation: an inflammatory phase (including the inflammatory immune response) over hours to days; a proliferation phase (including the anti-inflammatory immune response) over days to weeks; and a maturation phase (chronic low-level inflammation) over weeks to months [102]. During the inflammatory response immediately following MI, necrotic CMs release danger-associated molecular patterns (DAMPs) and cytokines, activating innate immune cells such as monocytes, neutrophils, and dendritic cells [103,104]. Reperfusion of the occluded artery also enhances leukocyte migration into the infarcted region. Neutrophils intensify tissue injury through increasing extracellular ROS and protease levels [105], while monocytes are rapidly polarized into inflammatory macrophages, which release proinflammatory cytokines and remove apoptotic cells [106]. After the apoptotic cells and other debris are cleared from the infarct area over the course of a day or more, macrophage polarization reprograms to an anti-inflammatory phenotype, which promotes angiogenesis, collagen deposition, myofibroblast proliferation, and wound healing [103,104]. The switch from a reactive macrophage response to one that is reparative marks the transition into the proliferative phase, named after the fibroblast response, to fill in tissue mass that had been CMs prior to MI. It is important to note that there is a dynamic shift within the immune response that takes time: dead cells must be removed, while at the same time the repair and scar formation processes are initiated [107,108]. The deposition of collagen to form scar tissue is a compensatory mechanism to preserve ventricular wall integrity and maintain heart function. However, as the maturation of scar tissue proceeds over the weeks and months post-MI, ongoing tissue stiffening, fibrosis, myofibroblast apoptosis, and cardiac remodeling may not be sufficient to preserve function, thus making the post-MI heart highly susceptible to heart failure [102].

The inflammatory response of the heart post-MI has important implications for the timing of clinical interventions, especially in the case of cell-based therapies. In the clinical trial Reinfusion of Enriched Progenitor cells And Infarct Remodeling in Acute Myocardial Infarction (REPAIR-AMI), the greatest improvement in cardiac function occurred when the implanted cells (bone marrow-derived cells, BMCs) were administered five or more days after a patient’s MI, which is past the peak inflammatory stage [109]. Preclinical studies show similar trends with the implantation of neonatal rat CMs in a rat cryoinjury model of MI, where cell engraftment increased with implantation in the granulation tissue phase (early anti-inflammatory phase with high vascular bed regeneration) or later [110,111]. Another important consideration for cell engraftment and the timing of intervention is the maturation of the scar. Although it is well known that the properties of the scar post-MI—including its size, location, composition, structure, and mechanics—influence the function of the heart, less is known regarding how scar maturation impacts SC-CM engraftment [112]. If a scar is beyond its deposition/remodeling phase, the modulation of the scar in the cardiac environment may be necessary to allow for implanted SC-CMs to make host connections and provide therapeutic benefits.

### 3.3. Persistent Hypoxia

Localized hypoxia that persists is a crucial characteristic to understand in the post-infarcted heart. As previously discussed, the heart is a highly aerobic organ, with a significant oxygen demand. Importantly, this oxygen demand cannot be met even after initial reperfusion through the macrovessel angioplasty of the occluded or narrowed coronary artery, as microvascular injury remains, resulting in prolonged, localized hypoxia [113,114,115]. A recent study using a fully automated 3D image analysis of a permanent coronary artery ligation porcine model found microvascular remodeling from 7 to 45 days post-MI, low blood flow capacity, long diffusion distances, and as a result, limited oxygenation post-MI [116]. Interestingly, there was an oxygen gradient across healthy tissue, the border zone, and the infarct, which was associated with changes in calcium handling, mechanical stresses, and inflammatory responses [117].

Given the prolonged hypoxia and accompanying pathological responses observed in the infarcted heart even after reperfusion, angiogenesis—the sprouting of new blood vessels from existing vessels—becomes increasingly important. Angiogenesis begins in the border zone and then extends into the infarct zone, forming new capillary beds [118]. Cell–cell communication has been shown to be central to the angiogenic response. Vascular endothelial growth factor (VEGF), platelet-derived growth factor (PDGF), and fibroblast growth factor (FGF) play important roles in several signaling circuits responsible for initiating angiogenesis in endothelial cells and vascular stromal cell populations [118,119]. Additionally, several intracellular signaling pathways are involved in the vascularization of ischemic tissue. Indeed, the PI3K/Akt/mTOR, Notch, Wnt/β-catenin, Hippo, Sonic Hedgehog, and JAK/STAT pathways are key regulators and potential therapeutic targets [119]. It is also important to note the relationships between HIFs (involved in other hypoxia responses), macrophages (involved in inflammation), and angiogenesis. Multiple in vivo studies have demonstrated that HIF-1α improves angiogenesis after MI. The administration of HIF-1α/VP16 had a similar therapeutic effect as VEGF in a rat MI model [120], and the overexpression of HIF-1α resulted in reduced infarct size, upregulation of VEGF and nitric oxide synthase expression, and improved capillary density in a mouse MI model [121]. Further, macrophage polarization (classically M1 pro-inflammatory and M2 pro-reparative) is known to influence the post-MI environment [122], with recent studies leveraging biomaterials [123,124], cytokines [125,126,127], and exosomes [128] to increase M2-like macrophages to increase angiogenesis post-MI. Despite these existing pro-angiogenic responses during cardiac remodeling, revascularization of the infarct zone remains limited, which is an important consideration for cell-based therapeutic intervention post-MI to ensure that implanted cells are adequately perfused at implantation (or shortly thereafter) or can withstand prolonged hypoxia.

## 4. Strategies for Maximizing SC-CM Therapeutic Regeneration

The leading therapeutic intervention in the field of cardiac regeneration is aimed at reintroducing cells lost due to MI by implanting stem cell (SC)-derived CMs. In this way, functional SC-CMs can “remuscularize” the heart and thus re-engineer cardiac function. In preclinical trials, injecting SC-CMs or implanting engineered cardiac tissues (ECTs) in animal models of MI has shown that heart function stabilizes or improves with remuscularization by up to 10% in LV ejection fraction (EF) [129,130,131,132,133,134,135,136,137]. Such studies have been translated from preclinical rodent models [129,130,131,132,138] to large animal models of MI in swine [135,136] and non-human primates [137,139]. Currently, human clinical trials are now underway using hESC cardiac progenitor cells in patches [140] or hiPSC-CMs in engineered tissues (NCT04396899) [141] in Europe; hiPSC-CMs in injected spheroids (NCT04945018) or implanted cell sheets (NCT04696328) in Japan; and injected hESC-CMs in the US (NCT05068674).

**Figure 6:**
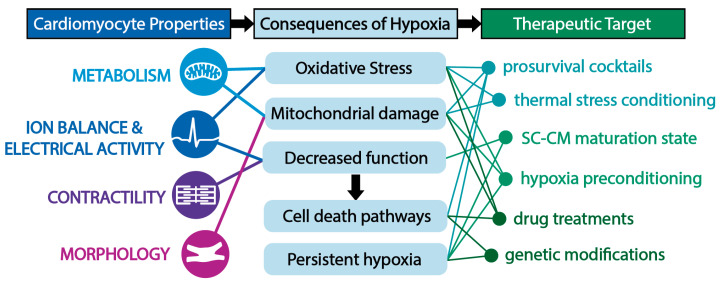
**Relationship between the experiences of CMs in hypoxia and strategies for maximizing SC-CM therapeutic regeneration.** The infarct zone remains hypoxic for days to weeks post-MI and reperfusion. Several features of CM pathophysiology (left, see Section 2) and consequences for the myocardium (middle, see Section 3) inform how novel therapeutics using SC-CMs can be modulated to improve cell survival and function in the heart’s hypoxic environment (right, see Section 4), with the ultimate goal of improving whole heart function with SC-CM-based cardiac regenerative therapies.

Ongoing research has focused on tuning the properties of SC-CMs to maximize their regenerative capacity in the harsh environment of the heart post-MI. It has been well documented that culture conditions and/or various treatments have the ability to impact SC-CMs in terms of structural organization [142,143,144,145], metabolic maturity [146,147,148,149,150], gene expression [144,151], and overall function [144,145,149]. Importantly, for the applications of SC-CMs as a regenerative therapy post-MI, consideration of the hypoxic environment into which SC-CMs are being implanted is vital in designing robust cell therapies that can survive this hostile environment, engraft into the host heart, and provide functional benefits. This section thus assesses the cell culture techniques and treatments that have been leveraged to tune the survival, maturation, and hypoxic resistance of SC-CMs (Figure 6). Although it is well known that the delivery mechanism (engineered tissue versus direct injection), use of biomaterials, and time point of intervention (e.g., acute vs. chronic intervention) can alter SC-CM engraftment into the heart through mechanical or other mechanisms (i.e., limiting SC-CM leakage, washout by the coronary circulation, and/or mechanical shearing), this review focuses on SC-CM treatments that directly alter their response to the hypoxia of the post-MI heart [152,153]. In this way, in vitro techniques that have shown promising results in terms of the SC-CM response to hypoxia (i.e., improved cell viability, maintenance/improved functional response, maintenance of normoxic gene expression, improved SC-CM engraftment post-MI, improved cardiac function upon SC-CM engraftment post-MI) are included in this review. We conclude this section by including genetic engineering approaches to accomplish this goal as well as others, namely immune acceptance by the host and the genetic control of cellular functions.

### 4.1. Development of Pro-Survival Cocktails

To enhance SC-CM survival upon transplantation into the post-MI heart, “pro-survival cocktails” have been developed and administered concurrent with SC-CMs to improve their survival. Such techniques are rooted in the pathophysiology of MI to inhibit mechanisms of cell death. An early example of this approach is from Laflamme et al., who, after observing the poor engraftment rate of human embryonic SC-CMs in infarcted hearts, developed a pro-survival cocktail that not only resulted in a persistent graft after 1 week but also increased the SC-CM graft size fourfold compared to their standard SC-CM treatment (heat shock 1 day prior to implantation and delivery of SC-CMs in Matrigel^®^) [129]. This pro-survival cocktail contained the following: Matrigel^®^ to prevent anoikis (i.e., cell death due to detachment); cyclosporine A and a peptide from Bcl-XL to block mitochondrial death pathways; pinacidil to open K^+^ channels, which mimics hypoxic preconditioning; insulin-like growth factor 1 (IGF-1) to activate the anti-apoptotic Akt pathway; and ZVAD-fmk to inhibit caspases associated with cell death. The combination of these compounds in the pro-survival cocktail was essential for its success in maximizing graft size. Other work has utilized miRNAs in cardiac pro-survival cocktails. For example, Hu et al. screened miRNAs in vitro, finding a potent pro-survival cocktail containing miR-21, miR-24, and miR-221 that improved the engraftment of cardiac progenitor cells in a mouse model of MI [154].

### 4.2. Thermal Stress Conditioning of SC-CMs

Under standard in vitro cell culture conditions, SC-CMs are maintained at 37 °C and 5% CO_2_. Modulating this environment within a physiological range has been shown to induce stress responses with significant genotypic and phenotypic changes within the cultured cells.

#### 4.2.1. Heat Shock

The heat shock response is a highly conserved biological process in which there is a rapid expression of proteins, known as heat shock proteins (HSPs), in response to elevated temperatures. In humans, elevated core body temperature, known as hyperthermia, is defined as above 40 °C (normal range is 36–37 °C) [155]. The role of HSPs is to serve as a defense mechanism against cellular stress through macromolecule stabilization, protein folding guidance, and misfolded protein removal [156]. HSPs can also reduce apoptosis through both intrinsic (i.e., preventing of the permeabilization of the mitochondrial outer membrane) and extrinsic (i.e., stabilizing the NF-κβ pathway) pathways [157]. In CMs specifically, HSPs have been shown to be induced under multiple conditions, including hyperthermia/heat shock, the presence of free radicals, cardiac stress, heart surgery, hypoxia, exercise, and environmental stress [156]. Further, the overexpression of HSP20 [158], HSP27 [159,160,161], HSP32 [162], HSP70 [163], and HSP72 [164] have been shown to decrease myocardial apoptosis during ischemia. The heat shock response has also been leveraged by cell-based cardiac regenerative therapies. For example, McGinley et al. overexpressed HSP27 in mesenchymal stem cells (MSCs) [165]. Upon implantation in a rat model of MI, MSCs overexpressing HSP27 had decreased apoptosis and led to a 1.8-fold increase in cell engraftment compared to control MSCs. Similarly, Wang et al. found HSP20 overexpression in MSCs improved cell survival at 4 days after injection into a rat model of MI [166]. In SC-CMs, Laflamme et al. demonstrated that heat shock one day prior to implantation increased the graft size of hESC-CMs three-fold when injected into healthy rat hearts [167], and thus heat shock pre-conditioning has since been broadly adopted in SC-CM implantation studies.

#### 4.2.2. Hypothermia

In contrast to heat shock (i.e., hyperthermia) is the use of hypothermia to improve the survival of CMs. Interestingly, hypothermia activates some of the same protective pathways as are targeted by hyperthermia. Using neonatal murine CMs, Shao et al. demonstrated hypothermia during ischemia–reperfusion (defined as 32 °C for the last 20 min of 90 min ischemia and first 1 h of 3 h reperfusion) decreased ROS production, maintained the mitochondrial membrane potential, and decreased cell death [168]. This enhanced CM protection at the cellular level was found to be mechanistically mediated by the increased generation of nitric oxide and the phosphorylation of Akt, a mitochondrial protection kinase regulated by HSP27 phosphorylation, during both the ischemia and reperfusion phases [168]. Chen et al. similarly found cellular protection in ischemia using a more moderate, yet extended hypothermia (33 °C, last 6 h of 12 h hypoxia and throughout 12 h reoxygenation). In this study, CM-like cell lines (H9c2, HL-1) were treated with CoCl_2_ to mimic hypoxia, resulting in decreased mitochondrial ROS; the increased expression of small ubiquitin-like modifier 1 (SUMO1) which plays an important role in post-translational protein modification during cellular stress; increased anti-apoptosis protein expression (Bcl-2); and decreased pro-apoptosis protein expression (caspase-3) [169]. In a recent study with both in vitro experiments using primary CMs in hypoxia and in vivo experiments using acute MI in mice, hypothermia at 32 °C promoted the increased expression of miR-483-3p, which served to downregulate cyclin-dependent kinase 9 (Cdk9) expression to reduce apoptosis, increase ATP levels, decrease cell death, and improve the contractility of the heart in vivo [170]. These studies demonstrate that several pathways activated by the hypothermia conditioning of SC-CMs include pro-survival stimulation and also modulate important cellular functions, like maintaining ATP and protein production.

### 4.3. Tuning the Maturation State of SC-CMs

SC-CMs display immature phenotypes compared to adult CMs in structure, metabolism, electrophysiology, calcium handling, and contractility [171,172,173,174,175,176]. RNA sequencing has been used to map the transcriptional landscape of SC-CMs during differentiation in vitro, finding that SC-CMs progress to a neonatal-like developmental stage in 2D culture in up to 30 days with standard culture conditions [151,177]. In the last decade, however, there has been significant progress towards maturing SC-CMs toward a more adult-like phenotype through a variety of techniques such as long-term culture [172]; culture in 3D constructs [178]; soft cell culture substrate and nanotopological cues [179]; mechanical loading [180]; electrical stimulation [181,182]; and metabolic conditioning [148]. Efforts to improve SC-CM maturation have a strong justification: matured SC-CMs more accurately recapitulate the adult cardiac physiology, which has been essential to eliciting known responses for validating drug screening models and revealing disease phenotypes in vitro [183,184]. In the context of heart regeneration, matured SC-CMs are theoretically more similar to the adult CMs of the host heart. Although maturation approaches, and thus the extent of the “maturation” achieved varies, “matured” SC-CMs do exhibit increased contractile stress generation and decreased arrhythmogenesis, which are important functional benefits of the implanted SC-CMS for the injured heart [174]. Of particular concern is the electrophysiological state of SC-CMs, as recent preclinical studies have found that arrythmias originating from human SC-CMs injected into the myocardial wall of pig and non-human primate hearts are sustained for 4 weeks before they resolve [134,135,137]. It is hypothesized that the electrophysiological immaturity of SC-CMs, in terms of increased automaticity, shorter actional potential durations, and depolarized mean resting membrane potential, likely contribute to the development of graft-initiated arrhythmias.

While the current mantra that SC-CMs are immature and need to be matured has some robust justification, particularly for in vitro studies, we posit that regenerative therapies are likely going to require a balance between SC-CM hypoxic resiliency and functional maturity. On one hand, it is important to consider the hypoxic environment of implanted SC-CMs due to the limited (if any) vasculature and the established pathology, such as ischemia, in the post-MI heart. Unlike healthy adult CMs that almost exclusively use oxidative phosphorylation, which produces a large amount of energy but requires a high level of oxygen, immature SC-CMs primarily rely on glycolysis, which cells can rely upon in low- or zero-oxygen environments.

Many different biophysical and biochemical approaches have been studied for advancing the maturation of SC-CMs, and the strong advancement of CM maturation metrics via the metabolic axis in recent years suggests that providing fatty acids for pushing SC-CMs towards an oxidative metabolic state may be the “golden ticket” to promoting a sufficiently mature SC-CM phenotype. However, a recent study by Peters et al. assessed the impact of maturing hiPSC-CMs through metabolic conditioning (i.e., high fatty acids), finding these cells had decreased viability and mitochondrial respiration in hypoxia compared to standard media (i.e., high glucose) [185]. This is well corroborated by recent work published from our own lab, in which a range of in vitro maturation techniques were systematically assessed in hiPSC-CMs in both 2D culture and 3D ECTs [186]. In this work, we found that hiPSC-CMs matured by altered metabolic substrates had significantly decreased contractile stress generation after hypoxia exposure compared to their glycolytically grown hiPSC-CM controls. An in vivo study by Reinecke et al. using fetal, neonatal, or adult rat CMs to form grafts in normal and injured hearts showed that only the fetal and neonatal CMs could form viable grafts and couple with the host myocardium [187], supporting the hypothesis that immature CMs have greater hypoxia resistance and thus increased engraftment success in the heart post-MI. So, these results beg the question of how to mature SC-CMs for translational heart regeneration while maintaining hypoxia resistance. Interestingly, Laflamme and colleagues combined the maturation techniques of the soft substrate culture (i.e., polydimethylsiloxane, ~400 kPa, compared to standard cell culture flasks of ~100 MPa) and long-term culture (40 days) of hiPSC-CMs, without altering the metabolic substrate, and reported improved graft electromechanical integration with the host heart, less proarrhythmic behavior, and a greater contractile benefit compared to standard (time-matched) hiPSC-CMs after injection into a guinea pig model of MI [179]. Such results point to the notion that SC-CM maturation occurs in a multi-factorial, networked manner, so that different techniques may be necessary to tune different maturation features within this network.

Regardless of the global SC-CM maturation state in vitro, however, it is important to note that SC-CMs undergo impressive maturation after implantation in vivo. Histological staining has been widely used to assess the structural changes SC-CMs undergo after implantation, finding an increased CM size [134], improved sarcomere development [134,135,137,188], and a transition from the expression of immature sarcomeric protein isoforms such as MLC2a and ssTnI to that of the more mature isoforms, MLC2v [132,135,188] and cTnI [137], respectively. In addition, SC-CMs implanted in vivo have increased expression and polarization of the adherens junction protein N-cadherin [134,135,167] and the gap junction protein connexin-43 [132,134]. Functionally, it has been well documented that implanted SC-CMs experience declined proliferation while in vivo [135,137,167,189], which is not surprising. There is also evidence of electrical maturation, as well as coupling with the host myocardium, as electrical quiescence and gap junction-mediated coupling to the host as shown by tachyarrhythmias declining to nearly zero after 4 weeks in vivo [135,137,190,191]. Although less is known about the mechanical maturation (i.e., active stress generation) of SC-CMs in vivo (currently due to technical challenges in assessing graft-specific contractile force in vivo), electromechanical integration will likely be vital to ensure the implanted SC-CMs contract in synchrony with the host heart to improve global metrics of heart function. Finally, transcriptomics have also shown that the gene expression of SC-CMs shifts toward a more mature genotype [188,189], with the increased expression of key cardiac maturation markers such as TNNI3, MYL2 (MLC2v), and MYH7 (β-MHC) [189]. It may be critically important to note, however, that the environment into which SC-CMs are being implanted appears to impact SC-CM maturation. For example, Halbach et al. found that SC-CMs implanted into the healthy myocardium, as opposed to the injured regions of the myocardium, resulted in the enhanced maturation of the SC-CMs shown by electrical activity and coupling with the host myocardium [192].

Future work will be required to determine an appropriate balance between an immature SC-CM phenotype that may prioritize hypoxia resistance and a mature SC-CM phenotype that may prioritize the electrical integration of the implanted SC-CMs and potentially a more rapid improvement in cardiac function. Targeting the specific features of SC-CM physiology that are impacted by hypoxia, such as metabolism, calcium handling, and mitochondrial dysfunction, in conjunction with maturation techniques focused on electromechanical function, is a potential approach that would allow for the maintenance of the functional benefits observed with SC-CM maturation while promoting cell survival and engraftment in the post-MI heart.

### 4.4. Hypoxia Preconditioning of SC-CMs

Hypoxia preconditioning (HPC), also referred to as ischemic preconditioning (IPC), involves one or more short periods of hypoxia/ischemia treatment prior to more severe or permanent injury. Murry et al. were the first to discover that HPC/IPC could significantly reduce infarct size and cell death in a dog model of MI [193], and these results have been replicated several times in other animal models and in humans [194,195]. HPC/IPC has also been studied at the cellular level, with several mechanistic pathways being implicated, such as the following: prolonged ATP production in hypoxia due to an increase in glycogen storage [196]; the upregulation of proteins involved in calcium handling to maintain calcium handling function [197,198]; the increased density of ion channels to combat their inactivation during prolonged ischemia [199]; mitochondrial stabilization, such as through the inhibition of MPTP opening [200]; and the activation of HIF-1 and nuclear factor erythroid-2-related factor 2 (Nrf2), which trigger a variety of cardioprotective proteins [201,202,203,204]. Such results indicate that the activation of certain pathways during hypoxia preconditioning mediates oxidative stress and apoptosis during prolonged hypoxia.

Despite the lack of clinical adoption of HPC/IPC on patients due to its risk and uncertainty in administration, such a technique can be easily adopted into current in vitro workflows [201]. For example, HPC/IPC has been used widely in the development of extracellular vehicles (EVs) as a cardiac therapy, with several groups demonstrating that EVs collected from hypoxic cells have a greater ability to preserve cardiac function after injury [205,206,207,208]. Although less is known about HPC/IPC adoption into the development of cell-based cardiac therapies, such a technique has the potential to be used as a potent trigger to induce a complex cascade of signaling to protect SC-CMs from prolonged hypoxia. In our own work, we found hiPSC-CMs in 2D as well as in 3D ECTs that underwent a single, short HPC treatment (30 min, 24 h prior to prolonged hypoxia exposure) could partially rescue the contractile stress generation of metabolically matured ECTs after hypoxia [186]. Further optimization of the timing, duration, and repetition of the hypoxia preconditioning period(s) may enable robust maturation and hypoxia resistance in SC-CMs for regenerative therapies.

### 4.5. Repurposing Drug Treatments for HIF Stabilization and Reducing Oxidative Stress

There are a range of drug treatments that have been developed to enhance the cellular response to hypoxic and metabolic stress, which is the basis for a variety of diseases. Through a cross-disciplinary approach, the efficacy of these developed drugs to enhance specifically the SC-CM response in hypoxia for cardiac regenerative therapies is being evaluated.

As described above, HIF-1α is a key regulator of the cellular hypoxia response and is a popular target for drug development, especially in cancer therapeutics, as hypoxia is a key feature of the tumor microenvironment that is leveraged by cancer cells to induce angiogenesis, activate proliferation, and adjust metabolism [209,210,211,212,213]. In the context of prolonged myocardial ischemia, HIF stabilization or activation could be desirable, given the role of HIF pathways in CM adaptation to hypoxia. Dimethyloxalylglycine (DMOG) is a prolyl hydroxylase inhibitor that stabilizes HIFs [214]. Ambrose et al. demonstrated that 24 h DMOG treatment of HL-1 CM-like cells resulted in a 43% decrease in oxygen consumption, compared to a 30% decrease in oxygen consumption in HL-1 CMs exposed to 24 h of hypoxia (2% O_2_) [214]. Furthermore, the study identified complex I in the electron transport chain and aconitase as being DMOG-dependent, and therefore HIF-dependent, while complex IV was HIF-independent [214]. Functional outcomes have also been achieved in studies using DMOG in animal models. Ockaili et al. found that rabbits treated with DMOG had a reduced infarct size and better recovery after MIRI [215]. Zhang et al. found that rats treated with DMOG had reduced fibrosis, apoptosis, and oxidative stress in the myocardium, which led to improved right ventricular function [216]. Importantly, DMOG treatment can partially mimic HPC/IPC by activating HIFs [217,218]. Other drugs that have been developed for other applications, such as molidustat and carvedilol, could be leveraged for inducing greater SC-CM hypoxia resistance. First, molidustat, which is currently being investigated in renal anemia/chronic kidney disease clinical trials, has been shown to stabilize HIF-1α and rescue metabolic dysfunction in hiPSC-CMs in vitro [219]. Furthermore, molidustat improved recovery post-MI in a type 2 diabetes rat model, which is especially notable given that insulin resistance blunts the cardiac hypoxia response [219]. Second, carvedilol, a third-generation beta-blocker currently used in heart failure patients, has recently been applied to cardioprotection during chemotherapy and to cardiac hypoxia. Carvedilol has been shown to decrease free radical release and apoptosis in doxorubicin-treated H9c2 cells [220], and to attenuate mitochondrial dysfunction in both H9c2 CMs and hiPSC-CMs treated with doxorubicin [221]. These results suggest that carvedilol has significant cardioprotective potential during chemotherapy, and importantly, that the molecular/cellular targets of carvedilol (i.e., mitochondrial function) are highly relevant to CMs in hypoxia. In fact, Hu et al. recently showed that the carvedilol treatment of murine-isolated CMs improved contractility and calcium handling in hypoxia by activating the AMPK signaling pathway [222]. Other drugs have also targeted the AMPK pathway during MI with reperfusion, including trimetazidine [223], empagliflozin [224], and dichloroacetate [225]. Therefore, drug repurposing is a feasible option for enhancing the regenerative potential of SC-CMs through multiple avenues, including HIF stabilization, maintenance of metabolism, and calcium and contractile dynamics.

### 4.6. Genetic Modification of SC-CMs

Whereas drug treatments of SC-CMs are well controlled in vitro but come with their own limitations (particularly used in vivo), the genetic modification of SC-CMs presents stable control over gene expression. For tuning the hypoxic response of SC-CMs through genetic modifications, Datta Chaudhuri et al. found that the overexpression of HIF-1α in hypoxic cardiomyocytes promoted the HO-1-induced antioxidant response, reduced ROS levels, and attenuated apoptosis [226]. Wang et al. demonstrated that the silencing of PHD2, which marks HIF-1α for degradation in normoxia, in human adipose-derived stem cells (ADSCs) implanted into the infarcted heart of mice, resulted in greater ADSC survival through a HIF-1α-dependent mechanism [227]. In addition to HIF, modulation of the Wnt signaling pathway has also been used to tune the SC-CM response to hypoxia. Wo et al. showed that β-catenin knockdown (i.e., Wnt inhibition) prevented hydrogen peroxide-induced damage in vitro and ischemia-induced damage in vivo [228]. Additionally, Aoyama et al. showed that expression of Wnt11 (a non-canonical Wnt protein) reduced CM necrosis in a myocarditis mouse model [229]. Given these results, the Wnt signaling pathway could be a potential target for the promotion of hypoxia resistance in hiPSC-CMs by preventing DNA damage and necrotic cell death.

In the field of cardiac regeneration, genetic editing holds promise for permanently altering the SC-CM response in vivo and in host–graft interactions. Notably, a more quiescent electrophysiological phenotype in hiPSC-CMs was engineered to curb graft-associated arrythmias upon their implantation in pig models through the silencing of depolarization-associated genes (HCN4, CACNA1H, and SLC8A1) and the overexpression of a hyper-polarization-associated gene (KCNJ2) [230]. More broadly, many groups are interested in creating “universal” SC lines that are either able to evade the host immune system or be recognized as a “self” and accepted by the host, precluding use of life-long immune suppressant drugs for maintaining grafts from implanted SCs [231]. Currently, genetic editing is being used to create SCs that are able to successfully evade the immune system through a variety of approaches including the following: inactivation of histocompatibility complex (MHC) class I and II genes and the overexpression of CD47 [232]; knock-out of the B2M gene, whose protein interacts with human leukocyte antigen (HLA) class I proteins [233]; as well as knock-in of cytotoxic T lymphocyte antigen 4 (CTLA4) and programmed death ligand-1 (PD-L1) [234]. Further, with the growth of synthetic biology and gene circuit control [235,236], the ability to alter specific cells, such as engrafted SC-CMs in the heart, using exogenous drugs as tools for gene expression control, may enable the engineering of SC-CMs to enhance their safety and function as SC-based therapeutics for the heart.

## 5. Conclusions

A multitude of changes take place at the cellular level during MI, including a shift in CM metabolism, ion imbalance, calcium overload, the disruption of electrical activity, loss of contractility, and morphological changes signaling irreversible cell injury and eventual death. Even after MI, the cardiac environment remains dynamic, inflammatory, and hypoxic for days to weeks after the initial event due to myocardial remodeling, multi-stage immune responses, and limited revascularization. Localized hypoxia in the infarct zone—and the implant location of cell-based therapies—is of the utmost importance given the highly aerobic nature of CMs. Modulating the response of implanted SC-CMs to the cardiac environment is crucial to maximize survival and engraftment, and thus the therapeutic benefits. This review provided an overview of the current mechanisms being employed in research to modulate SC-CM responses to implantation through the development of pro-survival cocktails, stress preconditioning including HPC, the tuning of the maturation state of SC-CMs, the use of novel drug treatments, and the genetic engineering of SCs and SC-CMs. Future work to guide these strategies (and their combination) toward the clinical translation of SC-CMs and to elucidate the underlying mechanisms promises to enhance the outcomes of SC-CM-based regenerative cardiac therapies.

## Data Availability

No new data were created or analyzed in this study.
